# Embryo Microinjection of Selenomethionine Reduces Hatchability and Modifies Oxidant Responsive Gene Expression in Zebrafish

**DOI:** 10.1038/srep26520

**Published:** 2016-05-23

**Authors:** J. K. Thomas, D. M. Janz

**Affiliations:** 1 Toxicology Graduate Program, University of Saskatchewan, Saskatoon, Saskatchewan, Canada S7N 5B3; 2 Toxicology Centre, University of Saskatchewan, Saskatoon, Saskatchewan, Canada S7N 5B3; 3 Department of Veterinary Biomedical Sciences, University of Saskatchewan, Saskatoon, Saskatchewan, Canada S7N 5B4

## Abstract

In previous studies we demonstrated that exposure to selenomethionine (SeMet) causes developmental toxicities in zebrafish (*Danio rerio*). The objectives of this study were to establish a dose-response relationship for developmental toxicities in zebrafish after embryo microinjection of Se (8, 16 or 32 μg/g dry mass of eggs) in the form of SeMet, and to investigate potential underlying mechanism(s) of SeMet-induced developmental toxicities. A dose-dependent increase in frequencies of mortality and total deformities, and reduced hatchability were observed in zebrafish exposed to excess Se via embryo microinjection. The egg Se concentration causing 20% mortality was then used to investigate transcript abundance of proteins involved in antioxidant protection and methylation. Excess Se exposure modified gene expression of oxidant-responsive transcription factors (nuclear factor erythroid 2-related factor *nrf2a* and *nrf2b*), and enzymes involved in cellular methylation (methionine adenosyltransferase *mat1a* and *mat2ab*) in zebrafish larvae. Notably, excess Se exposure up-regulated transcript abundance of aryl hydrocarbon receptor 2 (*ahr2*), a signalling pathway involved in the toxicity of dioxin-related compounds. Our findings suggest that oxidative stress or modification of methylation, or a combination of these mechanisms, might be responsible for Se-induced developmental toxicities in fishes.

Selenium (Se) is a well-documented teratogen in oviparous vertebrates including fishes, aquatic birds and amphibians^1^,^2^,^3^,^4^. As an essential trace element, adequate intake of Se is required to maintain physiological homeostasis in all animals, whereas a marginal increase in Se intake can lead to Se poisoning^1^,^5^. Studies in fish have reported a narrow margin of safety for dietary Se intake, and a steep dose-response relationship for developmental toxicities^1^,^3^,^6^. Anthropogenic activities such as mining, coal-based power production, oil refining, and agriculture can significantly increase mobilization of Se into aquatic systems^1^. The bioaccumulation and trophic transfer properties of Se are well documented, and such properties can cause persistent toxicities to fishes inhabiting Se-contaminated sites^7^,^8^. Selenomethionine (SeMet) is the dominant form of Se present in primary producers and consumers (approximately 50–70% of total Se) inhabiting aquatic ecosystems contaminated with Se^7^,^9^,^10^. During vitellogenesis, adult female fishes inhabiting Se-contaminated sites transport and deposit vitellogenin enriched in Se to their eggs, and such maternal transfer of excess Se can lead to developmental toxicities in early life stages of F1 generation fishes^1^. Although a number of maternal Se exposure studies have reported developmental toxicities in fishes, few of these studies attempted to investigate mechanisms of Se-induced developmental toxicities. Since maternal deposition of Se to ovarian follicles is highly variable, it is difficult to study mechanisms of Se-induced developmental toxicities in early life stages of fishes^2^,^11^. Microinjection techniques have been successfully used to deliver uniform doses of teratogenic chemicals in fish embryos^12^,^13^, and this approach has been extensively used in the field of mechanistic toxicology^14^,^15^.

Previous studies proposed a number of mechanisms for Se-induced developmental toxicities in early life stages of freshwater fishes. The first proposed mechanism of Se-induced developmental toxicities is altered protein function, which is due to non-specific insertion of SeMet in place of the essential amino acid methionine (Met) during protein synthesis^16^,^17^,^18^. Since both SeMet and Met have similar physico-chemical properties, SeMet is inserted into proteins in an unregulated, dose-dependent fashion^19^. However this mechanism is not fully accepted since previous studies demonstrated both impaired or normal function of proteins after exposure to excess SeMet^16^,^17^,^18^,^20^. The second and more accepted hypothesis for Se-induced developmental toxicity is oxidative stress^1^,^21^,^22^. Catabolism of SeMet is reported to produce redox-active intermediates such as methylselenol and selenide anion, and these metabolites are hypothesized to induce oxidative stress^21^,^22^. Developmental exposure to teratogenic chemicals in fishes has been demonstrated to induce oxidative stress and/or increase mRNA expression of oxidant-responsive transcription factors and enzymes^14^,^15^,^23^. Nuclear factor erythroid 2-related factor 2 (Nrf2) is an important transcription factor activated during oxidative stress^15^. In addition to the protein dysfunction and oxidative stress hypotheses, recent studies reported impaired DNA methylation in early life stages of zebrafish (*Danio rerio*) exposed aqueously to selenite^24^, and decreased transcript abundance of hepatic methionine adenosyltransferase 1A (*mat1a*) in adult zebrafish exposed chronically to elevated dietary SeMet^25^. Further research is needed to investigate the potential roles of oxidative stress and methylation status in early life stage toxicities resulting from *in ovo* SeMet exposure in fishes.

The objectives of the present study were to establish a dose-response relationship for mortality and deformities in early life stages of zebrafish after embryo microinjection of graded doses of Se in the form of SeMet, and to investigate potential underlying mechanism(s) of Se-induced developmental toxicities. There are many advantages of using zebrafish for developmental toxicology and embryo manipulation studies. Zebrafish produce transparent and non-adherent embryos which aids in successful microinjection of chemicals^26^,^27^,^28^. Since vast information is available on the developmental biology and genome of zebrafish, both developmental and mechanistic toxicological studies can be easily carried out in this fish species^27^,^28^.

## Results

### Concentrations of selenium

Measured and nominal Se concentrations were directly proportional in each treatment group. Measured concentrations of Se in control, 8, 16 or 32 μg Se/g dry mass (d.m.) microinjected groups were 1.6 ± 0.1, 11.0 ± 0.6, 18.7 ± 1.0, and 29.3 ± 0.5 μg Se/g d.m., respectively. A significantly greater concentration of Se was observed in eggs microinjected with 8, 16 or 32 μg Se/g d.m. when compared to eggs microinjected with Se-free Danieau solution (control) (*p* < 0.05; Table 1).

### Hatchability, mortality and total deformities

**Figure 1:**
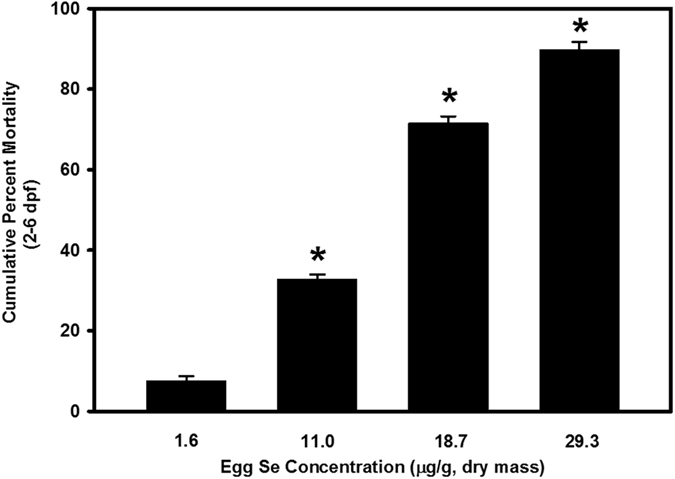
Percent cumulative mortality (2–6 days post fertilization [dpf]) of early life stages of zebrafish exposed to increasing concentrations of selenomethionine via embryo microinjection. *Significant difference compared to the control group using one-way ANOVA followed by Dunnett’s test (*p* < 0.05; *n* = 3–4 replicates of 30–45 embryos).

**Figure 2:**
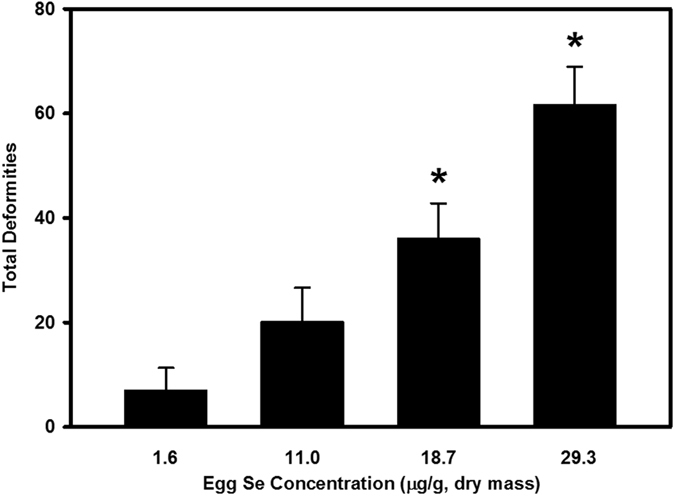
Total morphological abnormalities (sum of skeletal, craniofacial and fin deformities, and edema) in larval zebrafish exposed to increasing concentrations of selenomethionine via embryo microinjection. *Significant difference compared to the control group using one-way ANOVA followed by Dunnett’s test (*p* < 0.05; *n* = 3–4 replicates of 30–45 embryos).

**Figure 3:**
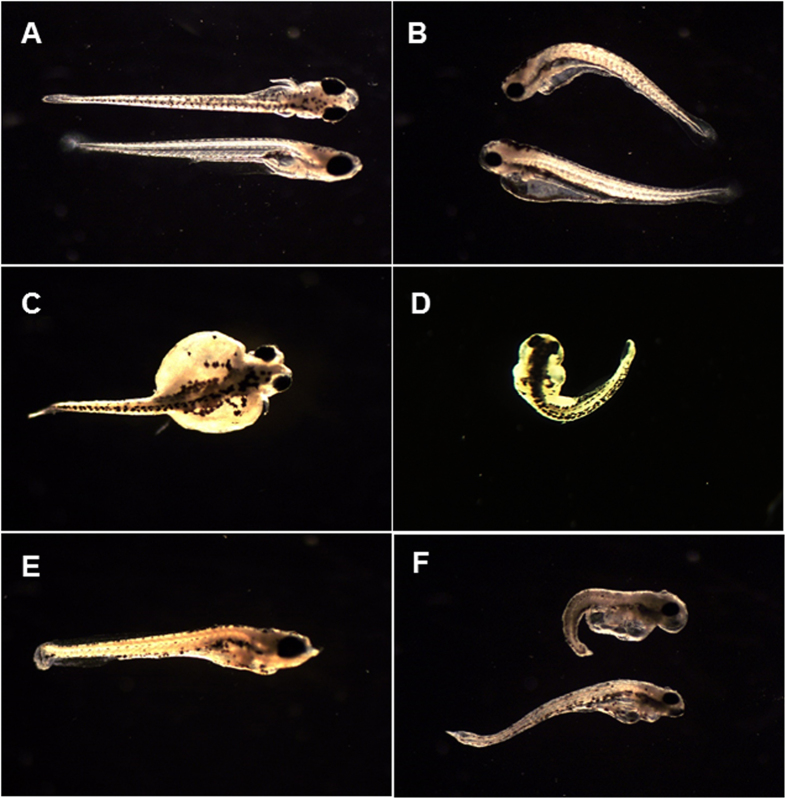
Images of normal (A) and deformed (B–F) larval zebrafish recorded during deformity analysis. Image **A** shows a normal (control) fish, **B** shows kyphosis and lordosis, **C** shows edema, **D** shows scoliosis, **E** shows fin deformities and **F** is a larval fish with multiple deformities and pericardial edema.

Mean embryo hatchability, cumulative percent mortality and total deformities of early life stages of zebrafish from the sham microinjected treatment group were not different from the control (Danieau solution-injected) group. Microinjection of excess Se in zebrafish embryos significantly reduced hatchability (*p* < 0.05; Table 1). Percent embryo hatchability was reduced from a mean of 94.2% in the control group to 75.1–19.9% in the SeMet-microinjected groups. Embryo microinjection of excess Se in the form of SeMet increased mortality and deformities of early life stages of zebrafish in a dose-dependent fashion (Figs 1 and 2). Significantly greater mortality was observed in early life stages of zebrafish from the 11.0, 18.7 and 29.3 μg Se/g d.m. treatment groups when compared to controls (*p* < 0.05; Fig. 1). Similarly, significantly greater deformities were observed in early life stages of zebrafish from the two highest Se-exposed treatment groups (18.7 and 29.3 μg Se/g d.m.) (*p* < 0.05; Fig. 2). Commonly observed malformations were skeletal curvatures (scoliosis, lordosis and kyphosis), fin deformities, craniofacial deformities, yolk sac edema, and pericardial edema (Fig. 3). Cumulative percent mortality increased from a mean of 7.5% in the control group to 32.8–89.8% in the SeMet-microinjected groups. Similarly, total deformities increased from a mean of 7.1% in the control group to 20.2–61.7% in the SeMet-exposed groups. Egg Se concentrations causing 10%, 20% and 50% mortality and deformities were 7.6, 9.6 and 14.9 μg Se/g d.m., and 9.0, 13.0 and 26.1 μg Se/g d.m., respectively.

### Effects of Se exposure on antioxidant responses

**Figure 4:**
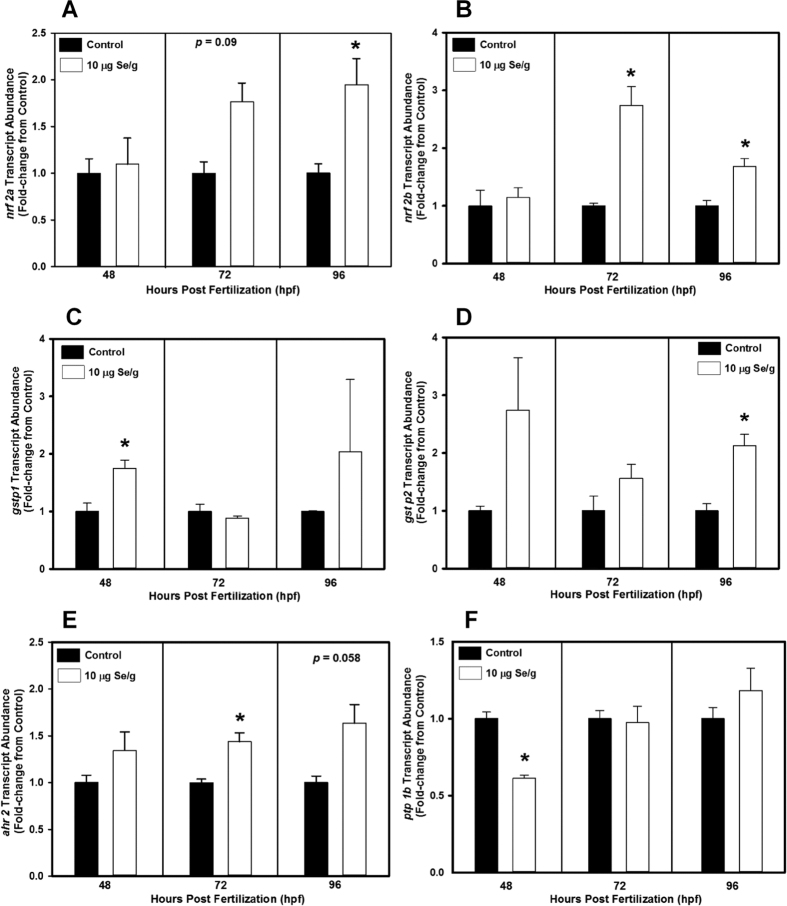
Transcript abundance of (**A)** nuclear factor erythroid 2-related factor 2a (*nrf2a*), (**B**) *nrf2b*, (**C**) glutathione S-transferase pi 1 (*gstp1*), (**D**) *gstp2*, (**E**) aryl hydrocarbon receptor 2 (*ahr2*), and (**F**) protein tyrosine phosphatase 1B (*ptp1b*) in zebrafish exposed to either 10 μg Se/g d.m. in the form of selenomethionine or control (Danieau) solution via embryo microinjection. Transcript abundance was determined by quantitative real-time PCR at 48, 72 and 96 hours post fertilization (hpf). *Significantly different from the control group using student *t*-test (*p* < 0.05; *n* = 3–5 replicates of 20 embryos and/or larval fish).

Transcript abundances of *nrf2a* and *nrf2b*, glutathione peroxidase 1a (*gpx1a*), glutathione S-transferase pi 1 and 2 (*gstp1* and *gstp2*), aryl hydrocarbon receptor 2 (*ahr2*), and protein tyrosine phosphatase 1b (*ptp1b*) were determined at 48, 72 and 96 hours post-fertilization (hpf) in zebrafish larvae from control and 10 μg Se/g d.m. microinjected groups. Up-regulation of *nrf2a* and *nrf2b* was observed at 72 and 96 hpf in zebrafish from the SeMet-exposed group when compared to the control group (Fig. 4A,B). Significantly greater up-regulation of transcript abundance of *nrf2a* was observed at 96 hpf, whereas transcript abundance of *nrf2b* was significantly up-regulated both at 72 and 96 hpf (*p* < 0.05; Fig. 4A,B). Expression of target genes downstream of *nrf2 (gpx1a, gstp1* and *gstp2*) were determined. Transcript abundance of *gpx1a* was not altered between treatment groups. However, significant up-regulation of *gstp1* at 48 hpf and *gstp2* at 96 hpf were observed in zebrafish from the SeMet-injected group compared to control (*p* < 0.05; Fig. 4C,D). Excess SeMet exposure of zebrafish via embryo microinjection significantly increased transcript abundance of *ahr2* at 72 hpf (*p* < 0.05), and there was a trend for increased expression at 96 hpf (*p* = 0.058; Fig. 4E). The transcript abundance of *ptp1b* was significantly lower at 48 hpf in early life stage zebrafish from the SeMet-exposed group when compared to control (*p* < 0.05; Fig. 4F).

### Effects of Se exposure on cellular methylation

**Figure 5:**
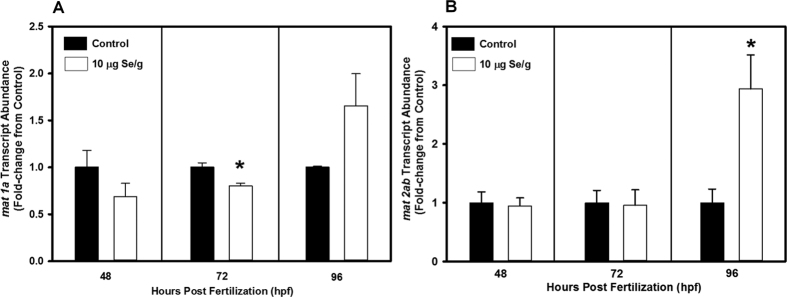
Transcript abundance of (**A**) methionine adenosyltransferase 1a (*mat1a*); and (**B**) *mat2ab* in zebrafish exposed to either 10 μg Se/g d.m. in the form of selenomethionine or control (Danieau) solution via embryo microinjection. Transcript abundance was determined by quantitative real-time PCR at 48, 72 and 96 hours post fertilization (hpf). *Significantly different from the control group using student *t*-test (*p* < 0.05; *n* = 3–5 replicates of 20 embryos and/or larval fish).

A significant down-regulation of transcript abundance of *mat1a* was observed at 72 hpf in the SeMet-microinjected group when compared to control (*p* < 0.05; Fig. 5A). Excess Se exposure significantly up-regulated transcript abundance of *mat2ab* at 96 hpf, however there were no changes in *mat2ab* observed at 48 and 72 hpf in zebrafish from the control and SeMet- microinjected groups (p < 0.05; Fig. 5B). There were no statistically significant differences in transcript abundances of *mat2aa* and *mat2b* between control and SeMet-injected groups (data not shown).

## Discussion

Maternal exposure to excess dietary Se (in particular SeMet) has been demonstrated to increase mortality and/or deformities in early life stages of F1 generation fishes^2^,^3^,^29^. The present SeMet embryo microinjection study was conducted with the intention to mimic maternal Se exposure, and was used to explore mechanisms of Se-induced toxicities in early life stages of zebrafish. The most notable findings of the present study were a significant reduction in embryo hatchability, a dose-dependent increase in mortality and deformities, and altered transcript abundances of *nrf2a, nrf2b, gstp2, ahr2, ptp1b, mat1a,* and *mat2ab,* in early life stages of zebrafish exposed to excess SeMet via embryo microinjection. Concentrations of Se used in this study were environmentally relevant and such concentrations have been reported in fish eggs collected from Se-contaminated aquatic ecosystems^11^,^29^,^29^,^30^.

In the present study, excess SeMet exposure in zebrafish via embryo microinjection caused a significant reduction in hatchability. This result was consistent with some previous studies where zebrafish or Japanese medaka (*Oryzias latipes*) embryos were exposed to waterborne selenite or SeMet^24^,^31^. However, the majority of maternal Se transfer studies in fishes have reported no significant changes in embryo hatchability^3^,^11^,^29^,^30^. Collectively, these results indicate that deposition of the free form of either organic or inorganic Se in eggs causes reduced hatchability in fishes. Previous studies have demonstrated maternal transfer of free amino acids from adult female fishes to their eggs^32^,^33^,^34^. Since SeMet is a seleno-amino acid with similar physico-chemical properties as the essential amino acid Met^19^, maternal transfer and deposition of free SeMet is possible in fish eggs. Previous studies have reported that embryonic fishes use both carbohydrates and free amino acids for energy production, whereas protein-bound amino acids and lipids are used for energy production only after hatching^32^,^33^,^34^. In addition, studies also suggest that embryonic fishes might preferentially retain free essential amino acids for protein synthesis^32^,^33^. Since SeMet mimics the essential amino acid Met, SeMet might be retained by embryonic fishes for protein synthesis. Thus, regardless of exposure route, embryonic fishes may use accumulated free SeMet for protein synthesis and/or energy production in a dose-dependent fashion. Catabolism of SeMet is reported to cause oxidative stress in developing fish embryos^21^, which may negatively affect embryo hatchability. Similarly, accumulation of SeMet in proteins has been reported to alter protein function^16^,^17^,^18^ and such protein dysfunction could also disrupt embryo development and cause subsequent embryo mortality or reduced hatchability. Collectively, oxidative stress due to catabolism of the free form of SeMet and/or SeMet-induced protein dysfunction might have caused the observed reduction in embryo hatchability in zebrafish in the present study.

Dose-dependent increases in mortality and deformities were observed in early life stages of zebrafish after embryo microinjection of SeMet. Greater mortality and/or deformities were also observed after microinjection of excess SeMet in pallid sturgeon (*Scaphirhynchus albus*)^35^, shovelnose sturgeon (*Scaphirhynchus platorynchus*)^35^ and white sturgeon (*Acipenser transmontanus*)^36^. In addition, several field and laboratory based maternal Se transfer studies also reported similar increases in mortality and/or deformities in F1 generation fishes^1^,^2^,^3^,^11^,^29^,^30^. In the present study, mortality was the most sensitive effect in early life stages of zebrafish rather than deformities. Similar to the present study, previous SeMet microinjection studies in white, pallid and shovelnose sturgeon also demonstrated that embryo-larval mortality was the most sensitive effect^35^,^36^. Conversely, the majority of laboratory and field based maternal Se transfer studies demonstrated that deformities were the most sensitive effect in early life stages of fishes^1^,^3^,^11^,^29^,^30^. The differences in Se-induced toxicities in early life stages of fishes via maternal transfer and microinjection routes might be due to the relative proportions of free versus protein-bound SeMet deposited or introduced to eggs by these routes. In the maternal transfer exposure route, the majority of Se deposition occurs as protein-bound SeMet, and lesser concentrations of free SeMet and other less toxic forms of Se (e.g., selenocysteine, seleno-proteins and inorganic Se) are also deposited in eggs. The protein-bound SeMet deposited in embryos via maternal transfer must undergo protein catabolism before being utilized for energy production and/ or protein synthesis. Studies have demonstrated that protein catabolism in early life stages of fishes occur only after hatching^32^,^33^,^34^. Hence, it is possible that protein-bound SeMet is utilized for energy production and/or protein synthesis at a later developmental stage than the free form of SeMet. Slow release of SeMet during protein catabolism might delay the onset and progression of Se toxicity in early life stages of fishes, and such slow progression of toxicity might cause deformities rather than mortality in early life stages of fishes. In the microinjection exposure route, the pure and free form of SeMet is introduced to embryos. Rapid utilization of the free form of SeMet by developing embryos for energy production and/or protein synthesis could cause quicker onset and progression of Se toxicity, thus preferentially causing mortality in early life stages of fishes. Collectively, these results suggest that SeMet might be the most toxic form of Se, and the ratio of free to protein-bound SeMet could possibly be used to determine the relative occurrence of deformities or mortality in early life stages of fishes.

The major focus of this study was to investigate mechanisms of Se-induced developmental toxicities in early life stages of fishes. Oxidative stress is one of the hypothesized mechanisms of Se-induced developmental toxicities in early life stages of fishes^1^,^21^,^22^. Catabolism of SeMet has been reported to produce highly reactive metabolites such as methylselenol and selenide anion, and these metabolites undergo redox cycling that may result in oxidative stress^1^,^21^,^22^. Developmental exposure to other teratogenic chemicals in zebrafish has been demonstrated to up-regulate transcript abundance of oxidant-responsive transcription factors (*nrf2*) and/or enzymes (*gpx1a* and *gstp*)^14^,^15^. Duplicate copies *nrf 2* genes (*nrf2a* and *nrf2b*) have been identified in zebrafish, whereas mammals have only one *nrf2* gene (*nrf2a*)^15^. Significant up-regulation of *nrf2a* and *nrf2b* in the present study suggests involvement of oxidative stress in Se-induced developmental toxicities in zebrafish. Previous studies have shown that the *nrf2* signalling pathway is involved in the activation of *gstp*^14^,^37^. Both up-regulation of *gstp2* and a trend for greater transcript abundance of *gstp1* in 96 hpf zebrafish exposed to excess SeMet in the present study suggests that such exposure can alter *nrf2* signalling pathways in early life stages of zebrafish. The present study did not observe significant up-regulation of transcript abundance of *gpx1a* in early life stages of zebrafish after exposure to SeMet via embryo microinjection. Similarly, a previous study also demonstrated no change in *gpx1* and greater transcript abundance of *gstp2* after aqueous exposure to SeMet in early life stages of zebrafish^38^.

To our knowledge this is the first study to provide evidence that exposure to Se can modify *ahr2* transcript abundance. SeMet exposure resulted in up-regulation of *ahr2*, suggesting that Se-induced developmental toxicities in zebrafish might also arise from the AhR2 signaling pathway (a pathway involved in toxicity of dioxin-like chemicals). A classic toxicity of dioxin-like chemicals is ‘blue sac syndrome’ or accumulation of fluids in the pericardial region of exposed early life stages of fishes^39^. Similar toxicity (i.e. pericardial edema) has been reported in a number of developmental Se exposure studies^2^,^38^,^40^,^41^, and Se-induced activation of the AhR2 signalling pathway may be involved in such toxicity. Both *nrf2a* and *nrf2b* have been demonstrated to participate in cross-talk with *ahr2* in zebrafish^15^. This bidirectional interaction might provide an explanation for the observed up-regulation of *ahr2* in the present study. Overall, our study suggests that oxidative stress along with up-regulation of *ahr2* may represent a mechanism of Se-induced developmental toxicities in zebrafish. Future studies should focus on whether co-exposure of SeMet potentiates toxicities of dioxin-like chemicals in early life stages of fishes. In addition, since *nrf2b* is identified as a negative regulator of expression of several genes during zebrafish embryo development^15^, it is of interest to investigate whether presence/up-regulation of this particular gene increases susceptibility of zebrafish to developmental Se toxicity. To date, no information is available on whether other teleost fishes possess two copies of the *nrf2* gene, and further studies are needed to determine whether other fishes possess an extra *nrf2* gene and whether or not presence of this gene makes teleost fishes susceptible to developmental Se toxicity.

There is a balance between the activities of protein tyrosine kinases (PTKs) and protein tyrosine phosphatases (PTPs) in cells that plays a pivotal role in cell signaling and animal development^42^,^43^,^44^. Oxidative stress and exposure to chemicals including SeMet have been demonstrated to inhibit transcript abundance and/or activity of protein tyrosine phosphatase 1B both *in vitro* and *in vivo*^25^,^42^,^45^,^46^. Previous studies demonstrated a role of *ptp1b* in cell-cell adhesion, angiogenesis, apoptosis and cell migration^43^,^47^,^48^, critical processes involved in embryo development in vertebrates. Hence, we investigated transcript abundance of *ptp1b* in early life stages of zebrafish after developmental exposure to excess SeMet via egg microinjection and observed that SeMet caused significantly down-regulated transcript abundance of *ptp1b* at 48 hpf. This finding suggests that modification of protein tyrosine phosphatase activity may also be involved in Se-induced developmental toxicities in zebrafish.

Exposure to excess Se (SeMet, selenocysteine or selenite) has been demonstrated to inhibit methionine catabolism or cellular methylation in animals^24^,^25^,^49^,^50^. These findings prompted us to investigate transcript abundance of genes involved in cellular methylation in early life stages of zebrafish after embryo microinjection of SeMet. Methionine adenosyltransferases are a family of enzymes that regulate the synthesis of s-adenosylmethionine (SAM), and SAM is an important substrate for methylation reactions in cells. Expressions of *mat1a, mat2aa, mat2ab* and *mat2b* have been reported in early life stages of zebrafish^51^. Significant down-regulation of *mat1a* at 72 hpf, and up-regulation of *mat2ab* at 96 hpf in early life stages of zebrafish exposed to SeMet suggests modification of cellular methylation. Developmental exposure to selenite in zebrafish has been shown to modify global DNA methylation^24^. Methylation is vital for cell growth, gene expression and biotransformation of toxicants including SeMet^52^,^53^,^54^,^55^. Modification of methionine catabolism, as indicated by down-regulation of *mat1a* and up-regulation of *mat2ab* in the present study, suggests modification of cellular methylation and/or SeMet detoxification. Impaired cell growth and gene expression, and inhibition of SeMet detoxification may negatively affect embryo or larval fish development and such effects could also cause developmental toxicities in early life stages of fishes.

In conclusion, our results suggest that deposition of greater concentrations of the free form of SeMet in eggs preferentially causes mortality rather than deformities in early life stages of fishes. Developmental exposure to excess SeMet via embryo microinjection altered gene expression of oxidant-responsive transcription factors, *nrf2a* and *nrf2b*, as well as *gstp1, gstp2, ptp1b, ahr2, mat1a* and *mat2ab*. These results indicate that developmental toxicities in excess SeMet-exposed zebrafish could be caused by oxidative stress or impaired methylation, or a combination of these mechanisms. Finally, our study indicates that embryo microinjection techniques can be successfully used to investigate mechanisms of toxicity of teratogenic chemicals in early life stages of zebrafish.

## Methods

### Test compound

Seleno-L-methionine was purchased from Sigma-Aldrich (Oakville, ON, Canada). Purity of the compound was greater than 98%.

### Test animal

All fish housing and experimental procedures adopted in this study were approved by the Animal Research Ethics Board at the University of Saskatchewan (protocol # 20030076), and adhered to the Canadian Council on Animal Care guidelines for humane animal use. Adult wild-type zebrafish (strain AB; approximately 4–5 months old) were purchased from a local supplier and housed in an environmental chamber with controlled temperature (28.0 ± 1.0 °C) and photoperiod (14 h light and 10 h dark). Mean wet body mass of fish was 0.366 ± 0.019 g. Adult fish were acclimated to laboratory conditions for 4 weeks prior to breeding. During the acclimation period fish were fed Nutrafin® basic flake food (Hagen Inc., Montreal, QC, Canada) and clean chironomids (Bio-Pure Blood Worms, Hikari Sales Inc., Hayward, CA, USA).

### Microinjection

Zebrafish breeding, maintenance and embryo manipulation were performed as previously described^26^,^56^,^57^. Immediately prior to microinjection, stock solutions of SeMet were diluted with Danieau solution^58^ to nominally achieve 8, 16 and 32 μg Se/g d.m. in embryos. Phenol red solution was added to injection solutions to monitor injection success. After the acclimation period, adult zebrafish were introduced in breeding tanks overnight (4 male: 8 female ratio). Embryos were collected the following morning and washed with E3 medium^56^ within 20–30 min after lights came on. Graded concentrations of SeMet or SeMet-free Danieau solution (control group) were injected at a constant volume of 3.14 nL/injection using a Narishige IM-300 microinjector (Narishige Laboratory Instruments Ltd., Tokyo, Japan) into the yolk region of embryos within 1.5–2 hours post-fertilization (hpf). A sham injection group was included in the experiment to investigate potential effects of embryo manipulation on hatchability, and incidence of mortalities and deformities in early life stages of zebrafish. After microinjection, embryos were incubated in Petri dishes with daily renewal of the E3 medium. Dead (opaque) eggs removed at 24 hpf from all treatment groups were considered not fertilized and removed from the experiment. Embryos were reared from 48–144 hpf to investigate effects of SeMet injection on embryo hatchability and frequencies of mortality and deformities in early life stages of zebrafish. Concentrations of total Se in eggs, percent hatchability, percent mortality and total deformities in larval fish were determined in all treatment groups as described previously^2^,^3^.

In addition, the egg Se concentration causing 20% mortality was calculated (~10 μg Se/g d.m.), and this dose was injected into yolk to investigate mRNA abundances of oxidant-responsive genes (*nrf2a, nrf2b, gpx1a, gstp1*, and *gstp2*), enzymes involved in methionine catabolism (*mat1a, mat2a, mat2aa* and *mat2ab*), *ahr2*, and *ptp1b* in 48, 72 and 96 hpf zebrafish.

### Deformity analysis

Total deformity analysis was carried out on 6 dpf larval zebrafish. The detailed deformity analysis procedure was explained previously^2^,^3^. Briefly, larval zebrafish were euthanized with an overdose of buffered tricaine methanesulfonate (MS-222) (Sigma-Aldrich, Oakville, ON, Canada) and preserved in 10% buffered formalin for 12 hours before being transferred to 70% ethanol. Each larval fish was examined for skeletal, craniofacial and fin deformities, and edema using an Olympus model SZ-CTV dissecting microscope (Olympus, Melville, NY, USA), and the presence or absence of developmental abnormalities was recorded for each fish. Total percent deformities were calculated by dividing number of malformed larval fish by total number of larval fish, and multiplying by 100.

### Quantification of selenium

Concentrations of total Se in pooled egg samples were measured by use of inductively coupled plasma-mass spectrometry (ICP-MS) at the Toxicology Centre (University of Saskatchewan, Saskatoon, SK, Canada). From each treatment group, *n* = 3–4 replicates of 45–50 pooled eggs were collected for quantification of Se. The detailed Se analysis procedure was described previously^3^,^59^. A limit of quantification (LOQ) of 0.13 μg Se/g was determined using method blanks. Concentrations of Se in eggs were measured on a wet mass basis, and converted to dry mass based on a moisture content of 92.5% determined in a subset of zebrafish eggs.

### Real-time polymerase chain reaction (Q-PCR)

Expression of mRNA for genes coding for enzymes or transcription factors of interest was quantified by use of quantitative polymerase chain reaction (Q-PCR). Total RNA was extracted from *n* = 3–5 replicates of 20 embryos and/or larval zebrafish from each treatment group using RNeasy Lipid Tissue Mini Kit (Qiagen, Mississauga, ON, Canada) according to the manufacturer’s instructions. Purified total RNA was quantified using a NanoDrop ND-1000 spectrophotometer (NanoDrop Technologies, Wilmington, DE, USA). A QuantiTect® Reverse Transcription Kit (Qiagen) was used to synthesis cDNA from 1 μg total RNA. Detailed procedures of RNA extraction and cDNA synthesis were explained elsewhere^25^.

Quantitative real-time PCR was performed in 96-well PCR plates by use of an ABI 7300 Real-Time PCR System (Applied Biosystems, Foster City, CA, USA). Gene specific primers were designed against target genes by use of Primer 3 software, and the sequences of primers are shown (Table 2). Detailed Q-PCR procedures were explained elsewhere^25^. Target gene transcript abundance was quantified by normalizing to the expression of elongation factor 1α (*ef1α*) according to the Mean Normalized Expression (MNE) method of Simon (2003)^60^.

### Statistical analysis

All statistical analyses were conducted using Sigmaplot 11 (Systat Software Inc., San Jose, CA, USA). Data were tested for normality by use of the Shapiro–Wilk test and for homogeneity of variance by use of Levene’s test. Data that did not meet the assumptions for parametric statistical procedures were log 10 transformed. Non-transformed data are shown in all figures. Significant differences in total Se concentrations in eggs, embryo hatchability, and mortality and total deformities of early life stages of zebrafish from the control and the graded SeMet microinjected groups were tested by use of one-way ANOVA followed by Dunnett’s test. Transcript abundances of antioxidant, methylation, AhR and protein phosphatase activity related genes in zebrafish from the control and 10 μg Se/g d.m. injected groups at 48, 72 and 96 hpf were tested by use of student *t*-test. Data were expressed as mean ± S.E.M. Differences were considered statistically significant at *p* < 0.05. Egg Se concentrations to cause 10, 20 and 50% mortality and deformities in early life stages of zebrafish were calculated by use of TOXSTAT® version 3.5 software (Western Ecosystems Technology, Cheyenne., WY, USA). Abbott’s formula was used to adjust mortality and deformities in the control groups.

## Additional Information

**How to cite this article**: Thomas, J. K. and Janz, D. M. Embryo Microinjection of Selenomethionine Reduces Hatchability and Modifies Oxidant Responsive Gene Expression in Zebrafish. *Sci. Rep.* **6**, 26520; doi: 10.1038/srep26520 (2016).

## Figures and Tables

**Table 1:** Nominal and measured selenium (Se) concentrations in eggs and hatchability of zebrafish exposed to selenomethionine via embryo microinjection.

**Nominal Se (μg/g, dry mass)**	**Egg Se (μg/g, dry mass)**	**Hatchability (%)**
Control	1.6 ± 0.1	94.2 ± 0.7
8.0	11.0 ± 0.6*	75.1 ± 4.6
16.0	18.7 ± 1.0*	51.8 ± 7.3*
32.0	29.3 ± 0.5*	19.9 ± 2.7*

Data are mean ± S.E.M of *n* = 3–4 replicates of 45–50 pooled eggs for quantification of total Se, and *n* = 3–4 replicates of 30–45 embryos for hatchability analysis.

^*^Significantly different from the control group using one-way ANOVA followed by Dunnett’s test (*p* < 0.05).

**Table 2:** Gene-specific primer sequences for the quantitative real-time PCR used in this study.

**Target**	**Accession #**	**Sequence (5′–3′)**	**Annealing temp.**
*ef1α*	NM_131263.1	F: CTTCAACGCTCAGGTCATCA	60
		R: CGGTCGATCTTCTCCTTGAG	
*ptp1b*	NM130924	F: CTTCACCGAGAGCATCACAA	60
		R: GTTCGTCGGGTTGTTCATTT	
*nrf2a*	NM_182889.1	F: ACACACACCTGAAGCAGACG	60
		R: GGCATCATGAGATCAGTGGA	
*nrf2b*	HQ661166.1	F: CCTGCCCAACAGACTCTCTC	60
		R: CGTCTTTGTCCGACTGTTCA	
*ahr2*	AF063446.1	F: CCAGAGCCCTACACAAGCAT	60
		R: TCCTTAAGTGGACGGTTTGC	
*gpx1a*	NM_001007281.2	F: GAAATACGTCCGTCCTGGAA	60
		R: CATAAGGGACACAGGGTCGT	
*gstp1*	NM_131734.3	F: TGGTGCTTTGAAGATCATGC	60
		R: CTGAAACAGCACCAGGTCAC	
*gstp2*	NM_001020513.1	F: GGACTGGATGAAGGGTGACA	60
		R: GCCTCACAGTCGTTCTTTCC	
*mat1a*	NM_199871.1	F: ATGCAGTTCTTGACGCACAC	60
		R: TGGTGTCTCGCACAATCTTC	
*mat2aa*	NM_001290080.1	F: TGACCGTTCAGCTGCTTATG	60
		R: GGGACGGAGGTCAAAGTTCT	
*mat2ab*	NM_001014296.1	F: TGCGACCAGATAAGTGATGC	60
		R: TCAAAGCCCTTGGTTGAGTC	
*mat2b*	NM_001013474.1	F: GATGCTCCCAATCCTTTGAA	60
		R: CAACCTTCTCCACCTCTCCA	
